# The moderating effect of participation in online learning activities and perceived importance of online learning on EFL teachers’ teaching ability

**DOI:** 10.1016/j.heliyon.2023.e13890

**Published:** 2023-02-18

**Authors:** Yongliang Wang, Ziwen Pan, Mingzhe Wang

**Affiliations:** a Associate Professor in Applied Linguistics, School of Foreign Languages and Cultures, Nanjing Normal University, Nanjing, China; b School of Foreign Languages, Henan University, Kaifeng, China; c School of Curriculum and Pedagogy, Faculty of Education and Social Work, The University of Auckland, New Zealand

**Keywords:** COVID-19, Online learning, Online education, EFL teacher, Teaching ability, Perception

## Abstract

With the sudden outbreak of COVID-19, many educational contexts shifted from traditional face-to-face instruction to online and remote modes of delivery. This inspired a surge of scholarly attention in various countries to disclose the status and perceptions of stakeholders regarding online education. However, most of the existing studies in second/foreign language contexts are limited to students’ and teachers’ perceived emotions and experiences in e-instruction. Moreover, the extent to which online participation and the perceived importance of e-education influence teachers’ teaching ability has been widely overlooked. To fill this gap, this study explored the moderating influence of EFL teachers’ participation in online learning activities and the perceived importance of online learning on their teaching ability. In doing so, a questionnaire was spread and filled in by 453 Chinese EFL teachers with different backgrounds. The results of Structural Equation Modeling (SEM) obtained by Amos (v. 24) indicated that individual/demographic factors do not affect teachers’ perceived importance of online learning. It was also demonstrated that the perceived importance of online learning and learning time does not predict EFL teachers’ teaching ability. Furthermore, the results reveal that EFL teachers’ teaching ability does not predict their perceived importance of online learning. However, teachers’ participation in online learning activities predicted and explained 66% of the variance in their perceived importance of online learning. The study has implications for EFL teachers and teacher trainers in that it improves their awareness of the value of technologies in L2 education and practice.

## 1 Introduction

The fast pace of internet and technology integration into various aspects of education has provided several affordances for academicians to form identities and transmit knowledge and expertise to larger groups of audiences [[1], [2], [3]]. In online classes, students and teachers have more freedom and equipment to learn, discuss, clarify, and progress considering curriculum objectives [4]. This electronic and remote mode of education was given a compelling rise with the outbreak of the COVID-19 pandemic which demanded a shift from traditional teaching [5]. During the pandemic, many educational systems all around the world had to devise online platforms and management systems that fit with physical distancing policies and instructional needs [6,7]. The drastic change forced teachers to develop their strategic planning and instructional methods in relation to the demands of a technology-driven education [8].

To obtain success and efficacy in teaching, many teachers, especially second/foreign language (L2) teachers, required professional development courses in which they were informed on new competencies, roles, and visions about remote education [9]. Consequently, teacher-student classroom interactions, communications, methodologies, identities, behaviors, and practices modified owing to the abrupt shift from face-to-face instruction to an online mode of delivery [[10], [11], [12], [13]]. Research indicated that an effective online instruction in L2 contexts needs technological infrastructures, functional software and platforms, professional development programs for teachers, positive attitudes, and willingness to accept technologies [14,15]. Other than these institutional and IT-related factors, L2 teachers’ perceived importance of online education and participation in online activities play a significant role in delivering an optimal and efficacious instruction [16]. Additionally, demographic factors and individual differences such as age, gender, and teaching experience level can determine the reluctance, quality, and outcome of online education [17]. However, most of the studies in this area have focused on the perceptions and emotions of L2 teachers and students during the pandemic [11,18,19] and the extent to which online participation and perceived importance of e-education influence teachers’ teaching ability has been widely overlooked. This area is significant for EFL teachers and online teacher education, as whole since teachers’ percpetions of online resources influences their instructional expertise, too. To fill this gap, the present study was an effort to unpack the moderating influence of EFL teachers’ participation in online learning activities and the perceived importance of online learning on their teaching ability. Moreover, the predictability of each of these constructs was examined and depicted in models extracted from Amos software (v. 24).

## 2 Background

### 2.1 Online education

With the rapid growth of innovative technologies, educational systems and institutions have shifted from traditional ways of education to online and digitalized ones [20,21]. Advancements in online delivery such as smart whiteboards, virtual reality, several applications, chatrooms, and learning management systems (LMS) provided opportunities for EFL teachers and students to form a virtual community of English language education [14]. Another compelling force was that of the COVID-19 pandemic, which imposed social distancing policies and distance learning all around the world. In this era, many universities and schools had to close their doors and switch from face-to-face education to online education [22]. In so doing, they were forced to provide various tools to foster internet access education must be highly digitalized. This form of instruction demanded new educational behaviors that fit with network interactive features [23]. According to Deng [24], online education can be useful in case it has some core characteristics, namely *openness* (granting everyone an equal learning opportunity and right), *extendibility* (collecting and disseminating several educational resources, teaching modes, technologies, and interactions from different contexts for different contexts), *flexibility* (being used anytime and anywhere with larger data bases), *intermediation* (mediating various educational tasks and activities via new technologies), and *manageability* (having interpersonal management during online instruction).

Before the pandemic, the use of e-learning was limited, yet the outbreak of a deadly disease pushed educators toward a new mode of instruction whose success and quality were difficult to judge given a scarcity of statistics [25]. Practically, online education obliged teachers and students to use both synchronous and asynchronous tasks and practices. In synchronous courses, students participated in interactive instructions that were technologically enhanced. However, asynchronous activities involved tests, projects, assignments, group discussion, reflections, and feedback [24]. Such activities were done through interactive video-based activities, online meetings and webinars, and keynote speakers [26]. Regardless of its nature, online education has empirically been found to incur numerous positive outcomes for education, in general [12,27,28]. However, other studies pointed to drawbacks of online education such as difficulties in keeping students’ attention, classroom management, participation, interaction, and preventing negative emotions such as negative attitude, stress, and boredom [12,18,[29], [30], [31]]. It is essential to note that the achievement of success in this new mode of instruction depends on several internal and external factors as explained below.

### 2.2 Teachers’ roles and competencies in online classrooms

In the context of e-learning and remote education, it is asserted that EFL teachers must take various roles in the process of teaching. In pioneering research, Berge [32] proposed four macro-categories of roles for online teachers encompassing 1) pedagogical role, 2) managerial role, 3) social role, and 4) technical role. In a similar manner, Berge and Collins [33] maintained that online teachers must take the role of a manager, filter, editor, facilitator, expert, marketer, helper, and discussion leader. In a more recent study, Martin et al. [34] introduced five roles for online instructors such as being a course designer, subject matter expert, facilitator, mentor, and content manager. Moreover Hung and Chou [35] listed assessment designer, discussion facilitator, course organizer, social supporter, and technology facilitator as crucial roles for an online teacher, too.

In order to take the mentioned roles successfully, teachers should have several competencies. Given the context and culture-based nature of competency, teachers may require dissimilar competencies [36]. However, the most important ones in online contexts include technological competency [37], assessment competency [38], and communication competency [39]. Additionally, they need to be competent in instruction and learning, technology use, and management and instruction [39]. Likewise, Moorhouse et al. [40] maintained that for online teachers, classroom management competencies, and interactional-technological competencies are critical. Similarly, Farmer and Ramsadale [41] proposed five competencies related to tools and technology, leadership and instruction, community and etiquette, active teaching, and instructional design. These studies indicate that teaching in online contexts is a challenging task that requires knowledge, practice, facilities, training, participation, and perceived importance so that one’s teaching generates success on the part of the learners.

### 2.3 The outcomes of online resources and milieus in L2 education

Given their close ties with the digital era, online resources that have exponentially increased during the outbreak of COVID-19 can bring about several outcomes for L2 students and teachers. A growing bulk of investigations has indicated that online contexts and resources have the potentiality to enhance L2 students’ writing performance [42], speaking skills [43], classroom interactions [44], and perceived classroom enjoyment [45]. Moreover, online education and resources have been reported to influence EFL students’ emotional states, especially reducing or stopping negative emotions like anxiety and boredom [11,12,18,46].

Considering teachers, research shows that online resources and platforms can improve their pedagogical effectiveness [47], translingual and collaborative practices [48], professional identity [49], interactional competence [40], and emotional experiences [50]. These outcomes and opportunities offered by online education inspired EFL teachers to take different roles and work on more competencies required in remote education. Given the contextual shift during the pandemic, EFL teachers had to develop their technological and pedagogical competencies at the same time.

### 2.4 Factors influencing teachers’ acceptance and implementation of online teaching

It is widely admitted that the success, acceptance, and effective implementation of online teaching and e-education through technologies depend on various factors internal and external to the teacher [34,51]. As pinpointed in the literature, teacher-related factors that considerably influence the acceptance and use of technologies and e-learning include technological literacy, technological pedagogical content knowledge (TPACK), attitudes, beliefs, motivation, habit, self-efficacy, performance expectation, and computer experience and skills [[52], [53], [54]].

Regarding external factors, research demonstrated that institutional structure, working culture, resources, social conditions, price value of technologies, feasibility of technology use, competitive advantage, and institutional readiness all determine the admission and employment of online education [5,52,54]. Moreover, the provision of professional development courses for teachers and their expected online resources and infrastructures influence their technology integration in their L2 classes [55,56]. In case these factors are considered and effectively dealt with, many positive outcomes may emerge in L2 education in an online space.

### 2.5 Related studies

With the emergence and spread of COVID-19 pandemic, numerous research studies were conducted on online education. In the context of L2 education delivered online, research indicates that students’ language learning is affected by online resources and instructions [57]. Likewise, an increasing body of research has focused on the emotions and experiences of EFL students such as their boredom, anxiety, coping strategies, enjoyment, pride, and self-confidence [11,12,18,46,47,58]. Other studies, however, highlighted the role of teachers’ perceptions and practices in e-education. For example, Xu, et al. [59] underscored the significance of self-confidence and the perceived importance of online teaching in language teachers' practices in online mode of delivery. Moreover, Cheung [60] examined L2 teachers’ use of technology in Hong Kong and maintained that their technological and pedagogical beliefs determine their implementation of online teaching.

In another study, Yuan and Liu [49] contended that L2 teachers’ identity shifts from imagined identity to pragmatic identity depending on the virtual space and resources. Likewise, Gao and Cui [61], examined teachers’ pedagogical beliefs about teacher roles and asserted that the use of online teaching activities depends on teachers’ beliefs about teaching. Other studies have highlighted teachers’ emotions and competencies such as technological, classroom management, and online teacher interactional competencies [40,62]. Moser and Wei [63] investigated L2 teachers retention in the job and argued that online teachers “felt untrained, marginalized, and emotionally overworked” (p. 26) during the pandemic. Another flourishing line of inquiry in this domain has focused on various pedagogical activities that may improve classroom interaction like telecommunication, virtual literature circles, telecollaboration, and intercultural projects [[64], [65], [66], [67]]. The required facilities and institutional supports for online education have also been widely studied [68,69]. Furthermore, in a recent study in China, Chen [70] considered L2 online teaching as an ecology and examined the link between teacher agency and digital opportunities. The results indicated that teacher agency was reinforced by digital tools, especially under the influence of teacher beliefs and social contexts.

These influential studies demonstrate that researching online education is by no means new in EFL contexts. Various aspects of teaching English in virtual spaces have been scientifically examined. However, the way EFL teachers with different backgrounds (age, gender, and experience) partake and perceive online learning activities and their impact on teaching ability has remained under-researched. This gap is significant in that the degree of importance that EFL teachers ascribe to learning activities and resources in online contexts can considerably influence their teaching beliefs, practices, and abilities. Inspired by this shortcoming in the literature, the present study examined the following research questions.

1. How much variance among Chinese EFL teachers using online learning resources in terms of their perceived importance of online learning can be predicted by individual/demographic factors such as age, gender, and teaching experience?
2. How much variance among Chinese EFL teachers’ teaching ability can be predicted by the perceived importance of online learning and learning time?
3. How much variance among Chinese EFL teachers’ perceived importance of online learning can be predicted by their teaching ability and factor influencing participation in online learning activities?

## 3 Method

### 3.1 Participants

A total of 456 teachers were recruited to participate in this study of 600 invited participants. Three participants' data were filtered out considering their reports of ages under 18 years old and their unified responses for all questionnaire items, leaving 453 valid data. The sample consists of 141 males (31.1%) and 312 females (68.9%) with ages ranging from 20 to 59 years old (*M* = 38.32, *SD* = 8.596). Participants also reported their teaching experience, subjects, level of education received, and teaching grades (Table 1).

**Table 1:** Demographic information of the participants.

Demographic Information Category	*N*	*%*
**Gender**		
Male	141	31.1
Female	312	68.9
Total	453	100
**Age**		
20–31	111	24.7
32–38	110	24.4
39–44	122	27.1
45–59	107	23.8
Total (Valid)	450	100
Missing Cases	3	
Total	453	
**Level of Education**		
Less than high school degree	1	.2
High school degree	1	.2
College degree	14	3.1
Bachelor's degree	366	80.8
Master's degree	68	15
Doctoral degree	3	.7
Total	453	100
**Teaching Subjects**		
Chinese literature	65	14.3
Math	62	13.7
English	179	39.5
Physics	25	5.5
Chemistry	21	4.6
Biology	29	6.4
Politics	20	4.4
History	16	3.5
Geography	16	3.5
Others	20	4.4
Total	453	100
**Teaching Experience (year)**		
0–10	167	36.9
11–21	149	32.9
22–40	137	30.2
Total	453	100
**Teaching Grades**		
1st to 2nd grade	16	3.5
3rd to 4th grade	8	1.8
5th to 6th grade	15	3.3
7th to 9th grade (middle school)	207	45.7
10th to 12th grade (high school)	207	45.7
Total	453	100

### 3.2 Instruments

Based on the objectives of the study, an adapted version of a scale developed by Bayar [71], which consists of three parts measuring 1) teachers' perceived importance of using online learning resources, 2) teachers' perceived teaching ability and 3) external factors that influence teachers' use of online learning resources. More specifically, drawing on Bayar’s [71], two scales were developed to measure the internal factors affecting teachers' participation and attitudes towards participating in professional development (Cronbach α = 0.96) and their self-efficacy (Cronbach α = 0.81). Moreover, to accommodate the need of the present research, we modified some words in each item to address teachers' perceived importance of using online learning resources. In this sub-scale, six items dealt with the perceived importance of the online learning scale that formed the first part of the questionnaire. Additionally, to measure teachers' self-efficacy, five items were created. The five-item scale was translated into Chinese without modification to preserve the scale validity. The reliability scores, as obtained by Cronbach α, were 0.940 for perceived importance of using online learning resources scale and 0.783 for perceived teaching ability scale.

Concerning external factors that influence teachers' use of online learning resources, Bayar [71] developed a scale consisting of five components with 25 items in total: time, funding, principal influence, colleagues influence, and school culture. The time factor focuses on the family and work responsibilities along with the schedule of professional development activities. The internal consistency score (α) generated from Bayar's work for the time factor was 0.643. Following that, the funding factor corresponds to evaluating how salary supplements affect teachers' intention to participate in professional development activities. The Cronbach α score for this component was .625 in Bayar's analysis. As for the principal influence, it stands for the influence of school principals' action on teachers' intention to participate in professional development.

It is worth noting that we applied a minor revision to situate the items in the Chinese elementary education context for the items in this factor. Specifically, due to the more prominent impact that teachers' direct supervisors may bring to them compared to school principals, we made a minor adjustment by adding the phrase "direct supervisor" (in Chinese) to the related items. For instance, instead of saying, "My principal expects me to participate in professional development activities,” we used "My principal and direct supervisors expect me to participate in professional development activities." Bayar observed a 0.869 Cronbach α score for this component. For the colleague influence, similar to the implication of the principal influence factor, this component measures the degree of change of teachers' intention to participate in professional development brought by the action of their colleagues. According to Bayar's work, the internal consistency score for this factor was 0.772.

Finally, the influence of school culture is represented by how school culture affects teachers' participation in professional development. For instance, the item like "In my school we share the belief that teachers can learn to improve student achievement" evaluates the value related to participating in professional development set by the school and its influence on teachers' behavior. Bayar witnessed a 0.741 internal consistency performance. For the consideration of situating the scale into the present research context, aside from the revision mentioned above for the principal influence factor, we also replaced the phrases like "professional development" with "online learning resources." We observed a 0.904 Cronbach α score for the five factors combined, signaling excellent reliability of the translated and revised scale.

### 3.3 Data collection procedure

In order to meet the objectives of this quantitiave study, first, the instruments were meticulously examined and some modifications were made to items and their wording to measure the constructs of concern in the study. After ensuring the psychometric properties of the questionnaire, which was in the form of a booklet including three sub-scales (i.e., teachers' perceived importance of using online learning resources, teachers' perceived teaching ability, and external factors that influence teachers' use of online learning resources), an online data collection method was adopted with convenient sampling strategy. To gather the data, the questionnaire was imported into the Wenjuanxing platform, which is a popular Chinese online website that excels in designing and distributing questionnaires. Once the questionnaire design was completed, a web link and a QR code embedded in a poster were generated.

A total of 456 participants (out of 600) completed the questionnaire (return rate = 76%) by either scanning the QR code or accessing the link. In doing so, they were provided with detailed instructions for each part of the instrument regarding how to respond to the items. Prior to accessing the instrument, participants were presented with a consent form, informing their rights and that their identity would remain anonymous. Only those, who chose to participate in the study voluntarily, were granted access to the instrument, and their data were preserved for analysis. Moreover, we offered a small tribute of 3 RMB to participants upon completion of the questionnaire as our appreciation for their participation. The data collection procedure took three months, from December 2021 to February 2022.

### 3.4 Data analysis

In order to analyze the collected data, in this study, the researchers first examined the reliability of the scale through Cronbach’s Alpha coefficient. Then for the first and the second research questions, models were extracted from the data using Amos software. Moreover, Chi-square Value and Root Mean Square Error of Approximation were calculated. However, in the third research question, other than model extraction, model fit, Chi-square Value, and Root Mean Square Error of Approximation, the researchers used Regression Weights to calculate the amount of variance explained by variables.

## 4 Results

### 4.1 Results for the first research question

**Fig. 1:**
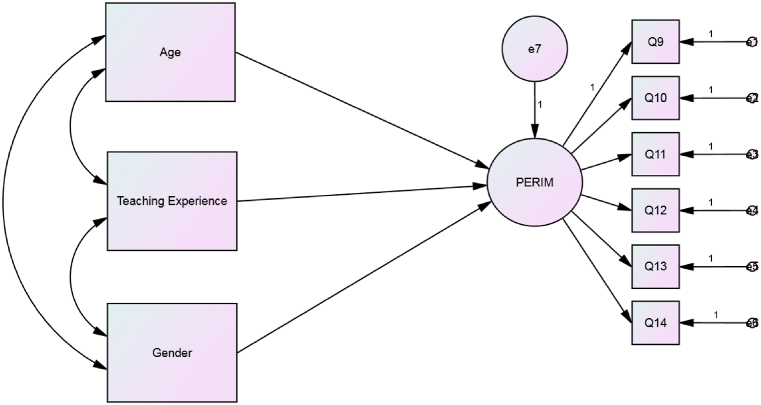
The research model for the first research question.

At the outset of the study, to check the reliability of the questionnaire, the final version of the questionnaire was piloted with 40 participants of the same population. The model diagram is shown in Fig. 1. Using Cronbach’s Alpha coefficient, it showed the reliability index of 0.87 (r = 0.87) for the Questionnaire. The results of this analysis are presented in Table 2.

**Table 2:** The results of reliability analysis of three questionnaires.

Questionnaire	Cronbach’s Alpha	Cronbach’s Alpha Based on Standardized Items	N of Items
Teachers' Use of Online Education Resources	.876	.874	44

**Fig. 2:**
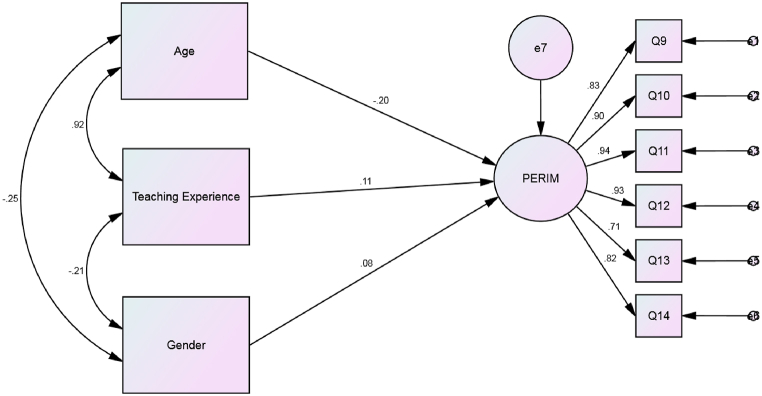
Model does not fit in the standardized estimation mode for the first research question.

The model was not fitted in Amos 24 software (see Fig. 2). The results were obtained as follows.

The software output (Chi-square = 302.006, Degrees of freedom = 24, and Probability level = .000) indicates that Chi-square test is significant (Sig = 0.000 < 0.05), so it can be concluded that there is a significant difference in the frequency of variables.

CMIN stands for the Chi-square value and is used to compare if the observed variables and expected results are not statistically significant. In other words, CMIN indicates if the sample data and hypothetical model are not an acceptable fit in the analysis. The value of interest here is the CMIN/DF for the default model and is interpreted as follows: If the CMIN/DF value is ≤ **3** it indicates an acceptable fit. The results of Table 3 show that the value for CMIN/DF is 12.584 that is not less than 4.

**Table 3:** The results of chi-square value.

Model	NPAR	CMIN	DF	P	CMIN/DF
**Default model**	21	302.006	24	.000	12.584
**Saturated model**	45	.000	0		
**Independence model**	9	3738.691	36	.000	103.853

RMSEA stands for Root Mean Square Error of Approximation and measures the difference between the observed covariance matrix per degree of freedom and the predicted covariance matrix. Root Mean Square Error of Approximation where values higher than 0.1 are considered poor, values between 0.08 and 0.1 are considered borderline, values ranging from 0.05 to 0.08 are considered acceptable, and values ≤ 0.05 are considered excellent. The results of Table 4 reveal that the RMSEA for the study is 0.160 that is considered as a poor value. It means that age, gender, and teaching experience do not predict EFL teachers using online learning resources in terms of their perceived importance of online learning. In other words, individual factors do not affect teachers’ perceived importance of online learning.

**Table 4:** The results of root mean square error of approximation for the first question.

Model	RMSEA	LO 90	HI 90	PCLOSE
**Default model**	.160	.144	.176	.000
**Independence model**	.475	.463	.488	.000

### 4.2 Results for the second research question

**Fig. 3:**
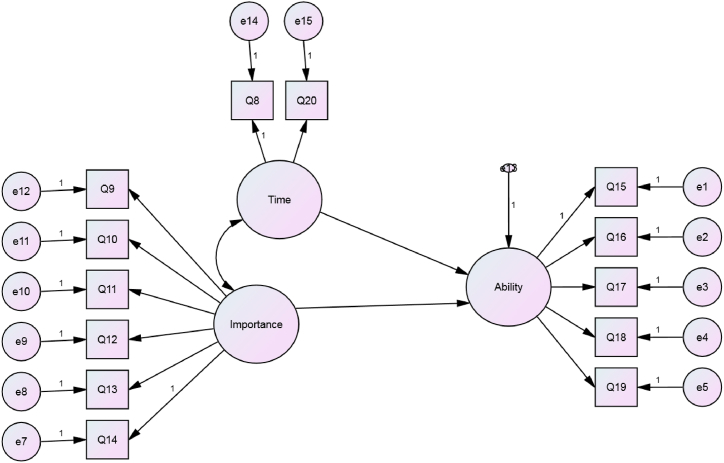
The research model for the second research question.

In order to answer this research question that focused on revealing how much variance among EFL teachers’ teaching ability can be predicted by perceived importance of online learning and learning time, a model was developed and tested for fitness via Amos 24 software (Fig. 3).

**Fig. 4:**
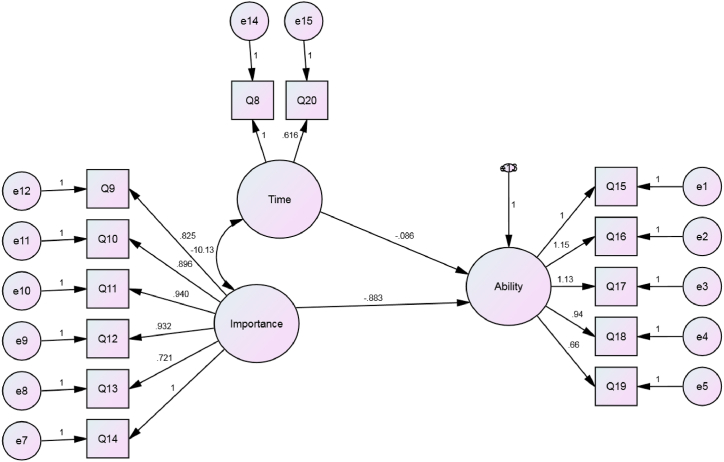
Model does not fit in the standardized estimation mode for the second research question.

The model was not fitted in Amos 24 software (see Fig. 4). The results were obtained as follows.

The software output (Chi-square = 517.018, Degrees of freedom = 62, and Probability level = .000) indicates that Chi-square test is significant (Sig = 0.000 < 0.05), so it can be concluded that there is a significant difference in the frequency of variables.

CMIN stands for the Chi-square value and is used to compare if the observed variables and expected results are not statistically significant. In other words, CMIN indicates if the sample data and hypothetical model are not an acceptable fit in the analysis. The value of interest here is the CMIN/DF for the default model and is interpreted as follows: If the CMIN/DF value is ≤ **3** it indicates an acceptable fit. The results of Table 5 show that the value for CMIN/DF is 8.339 that is not less than 4.

**Table 5:** The results of chi-square value for the second question.

Model	NPAR	CMIN	DF	P	CMIN/DF
**Default model**	29	517.018	62	.000	8.339
**Saturated model**	91	.000	0		
**Independence model**	13	4074.898	78	.000	52.242

The results of Table 6 reveal that the RMSEA for the study is 0.127 that is considered as a poor value. It means that the perceived importance of online learning and learning time does not predict EFL teachers’ teaching ability. In other words, the perceived importance of online learning and learning time do not affect EFL teachers’ teaching ability.

**Table 6:** The results of root mean square error of approximation for the second question.

Model	RMSEA	LO 90	HI 90	PCLOSE
**Default model**	.127	.117	.137	.000
**Independence model**	.336	.327	.344	.000

### 4.3 Results of the third research question

**Fig. 5:**
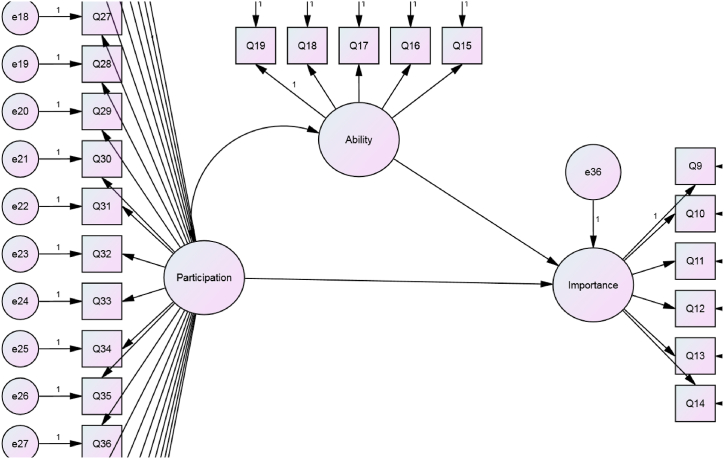
The research model for the third research question.

In response to this research question, which concerned the degree to which variance among EFL teachers’ perceived importance of online learning can be predicted by their teaching ability and factor influencing participation in online learning activities, as the previous question, the researchers designed a model, which was then tested for fitness using Amos software (v. 24) as presented in Fig. 5.

**Fig. 6:**
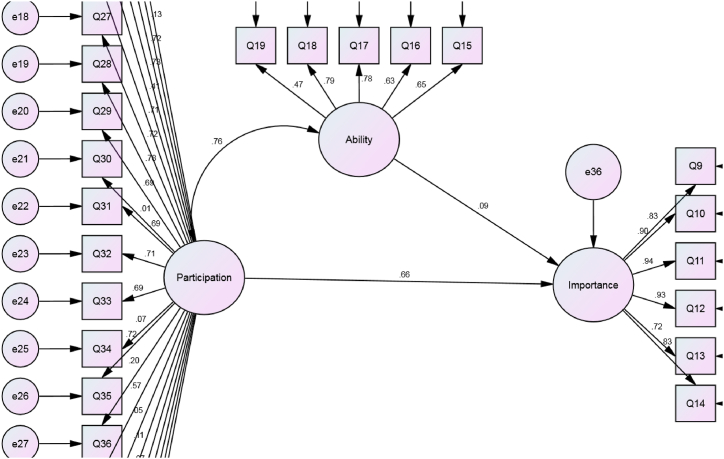
Model does not fit in the standardized estimation mode for the third research question.

As illustrated in Fig. 6, the model was not fitted in Amos 24 software. The results were obtained as follows.

The software output (Chi-square = 3335.878, Degrees of freedom = 557, and Probability level = .000) indicates that Chi-square test is significant (Sig = 0.000 < 0.05), so it can be concluded that there is a significant difference in the frequency of variables.

The value of interest here is the CMIN/DF for the default model and is interpreted as follows: If the CMIN/DF value is ≤ **3** it indicates an acceptable fit. The results of Table 7 show that the value for CMIN/DF is 5.989 that is not less than 4.

**Table 7:** The results of chi-square value for the third question.

Model	NPAR	CMIN	DF	P	CMIN/DF
**Default model**	73	3335.878	557	.000	5.989
**Saturated model**	630	.000	0		
**Independence model**	35	11070.330	595	.000	18.606

The results of Table 8 reveal that the RMSEA for the study is 0.105 that is considered as a poor value. It means those EFL teachers’ teaching ability and factor influencing their participation in online learning activities do not predict EFL teachers’ perceived importance of online learning. However, the results show that EFL teachers’ participation in online learning activity can predict their perceived importance of online learning. The prediction estimates are presented in Table 9.

**Table 8:** The results of root mean square error of approximation for the third question.

Model	RMSEA	LO 90	HI 90	PCLOSE
**Default model**	.105	.101	.108	.000
**Independence model**	.197	.194	.200	.000

**Table 9:** Estimates of regression weights for the variables in third question.

		Estimate	S.E.	C.R.	P	Label
**Perceived Importance**	**<---Teaching Ability**	.191	.145	1.318	.188	par_33
**Perceived Importance**	**<---Teachers’ Participations**	.663	.072	9.207	***	par_34

The results of Table 9 confirm that EFL teachers’ participation in online learning activity and EFL teachers’ teaching ability made contributions to EFL teachers’ perceived importance of online learning. However, EFL teachers’ participation in online learning activity made statistically significant contributions to variances in EFL teachers’ perceived importance of online learning. The results of Table 9 indicate that EFL teachers’ participation in online learning activity uniquely explains about 66% of the variance in EFL teachers’ perceived importance of online learning.

## 5 Discussion

The present study was an attempt to figure out the moderating influence of EFL teachers’ participation in online learning activities and the perceived importance of online learning on their teaching ability. Moreover, the predictive power of these constructs as well as the impact of background/demographic factors were examined. The results revealed that age, gender, and teaching experience do not predict EFL teachers using online learning resources in terms of their perceived importance of online learning. In other words, individual/demographic factors do not affect teachers’ perceived importance of online learning. The results are in conflict with those of Carril et al. [17] and Martin et al. [34], who argued that teachers’ demographic factors including age, gender, and prior teaching experience influence their attitude toward online education, perceived importance of this mode of delivery, and pedagogical practices. However, the findings partially support those obtained by Bayer [71], who maintained that teachers’ gender, teaching experience, and education level do not affect their participation in professional online activities. A possible reason for the obtained finding in this study that indicated no influence for demographic factors can be the idea that the EFL teachers in this study, regardless of their background, equally considered online learning and education important. The rapid shift toward e-learning during the outbreak in China can also be a reason behind the finding. In other words, during the pandemic all EFL teachers had no option but to work with remote and e-education. Hence, background factors played little or no role in their perceptions.

In this study it was demonstrated that the perceived importance of online learning and learning time does not predict EFL teachers’ teaching ability. In other words, the perceived importance of online learning and learning time does not affect EFL teachers’ teaching ability. This finding is in contrast with that of Xu et al. [59], who endorsed the powerful impact of teachers’ perceived importance of online teaching in their classroom practices and teaching skills in an online mode of delivery. Likewise, the results are inconsistent with Bayer [71], who ran a study in Turkey on teachers and found that learning time is a statistically significant factor in determining teachers’ pedagogical practices. The findings of the present research can be attributed to the sudden rush toward e-education in China during the pandemic, which minimized the influence of factors like perceived importance and learning time on EFL teachers’ teaching ability. It seems that the participants of this study comparably assigned importance to online instruction regardless of time. They may have had similar teaching abilities in a way that they all considered e-education of equal value and significance.

In this study, it was also shown that EFL teachers’ teaching ability does not predict EFL their perceived importance of online learning. It means that the level of expertise in teaching has no impact on their perceived degree of importance in online instruction. This is in conflict with Antwi-Boampong [52], who argued that the acceptance and implementation of online modes of education depend on teachers’ teaching skills as well as technological literacy. A reason for the obtained results can be the EFL context of China in which the teachers seem to feel an equal importance for online instruction regardless of their teaching ability. During the pandemic, all Chinese teachers as well as those all around the globe had to accept and work with a new mode of instruction. Hence, their perceived importance had no role to play. Both novice and experienced/expert teachers seem to equally consider online learning critical at the time of the study.

The results also indicated that EFL teachers’ participation in online learning activities (professional development courses) predicts and explains 66% of variance in their perceived importance of online learning. This finding is in line with Kohnke and Moorhouse [9] and Ghateolbahra, and Samimi [55], who asserted that professional development courses and online activities for teachers influence their perceptions of technology integration in their L2 classes. This can be because of the Chinese participants’ perceived need for professionalism and resources regarding online instruction. Given these needs and expectations, they may have considered online education important in a similar way. Another reason can be the complications that emerged during the pandemic that inspired many EFL teachers to demand and attend online courses regarding teaching English online. All these factors led to a high importance perceived by L2 teachers.

## 6 Conclusion and implications

According to the obtained results in this quantitative study, it can be concluded that individual/demographic factors do not affect teachers’ perceived importance of online learning. This is largely dependent on the context and teaching circumstances that shape the acceptance and perceived importance of online instruction on the part of EFL teachers. Moreover, it can be claimed that L2 teachers’ teaching ability and learning time in the context of e-education play a limited (if any) role in forming their perceived importance. Since the pandemic forced all teachers to shift from traditional mode of delivery to a remote one, many factors lost their impact. The main goal of e-instruction was to find a way to preserve education during an outbreak. As a result, several teachers demanded and attended professional development courses to become familiar with various online resources and activities that determine their pedagogical practices and perceived value of e-education. Following a similar trend to unpack the status e-learning and the perceptions of EFL teachers regarding online learning resources, the present study can offer some implications for different stakeholders in EFL contexts.

First, EFL teachers can use the results of this research in that they get familiar with the importance of their perceptions and practices of technologies in L2 education. They may use different activities and practices by which the integration of technologies in the classroom fosters students’ language learning. Second, teacher trainers can benefit from this piece of research by proposing courses to novice, experienced, pre-service, and in-service EFL teachers in which various aspects of online education are explicitly taught. During complex situations, as the pandemic, EFL teachers require practical courses regarding technology-integration as a way to improve and facilitate both teaching and learning. Third, curriculum designers can use this study to offer courses in which the role of technology in L2 education is highlighted. Finally, researchers may find this study beneficial by running more studies on the variables covered. They can compensate for the limitations of this study. For example, instead of following a mere quantitative research design, as the present study, future researchers can use mixed-methods, qualitative, and case studies to provide deeper insights. A balanced number of participants regarding their gender can be sought out in future studies. In this study, the number of females was greater than males. However, since ‘gender’ was not a variable of concern in the analyses, it did not influence the extracted models. Yet, future reserachers can avoid from pitfalls of unbalanced genders. The mediating role of cultural differences in perceiving e-education as important and implement it in L2 classes is also a new line of research. Finally, the extracted models in this study can be replicated in other EFL contexts with larger sample sizes to see if they change across educational contexts.

## Author contribution statement

Yongliang Wang: Analyzed and interpreted the data; Contributed reagents, materials, analysis tools or data; Wrote the paper.

Ziwen Pan: Conceived and designed the experiments.

Mingzhe Wang: Performed the experiments; Contributed reagents, materials, analysis tools or data.

## Funding statement

This work was supported by 10.13039/501100004609Foundation of Education Department of Henan Province [2022-JSJYYB-027].

## Data availability statement

Data will be made available on request.

## Declaration of interest’s statement

The authors declare no competing interests.
